# A conserved female-specific larval requirement for *MtnB* function facilitates sex separation in multiple species of disease vector mosquitoes

**DOI:** 10.1186/s13071-021-04844-w

**Published:** 2021-06-26

**Authors:** Keshava Mysore, Longhua Sun, Joseph B. Roethele, Ping Li, Jessica Igiede, Joi K. Misenti, Molly Duman-Scheel

**Affiliations:** 1 Department of Medical and Molecular Genetics, Indiana University School of Medicine, Raclin-Carmichael Hall, 1234 Notre Dame Ave., South Bend, IN 46617 USA; 2 University of Notre Dame Eck Institute for Global Health, Notre Dame, IN USA

**Keywords:** *Aedes aegypti*, *Aedes albopictus*, *Anopheles gambiae*, *Culex quinquefasciatus*, Sex, Larvae, Methionine, *Saccharomyces cerevisiae*, Yeast, Larvicide, RNAi

## Abstract

**Background:**

Clusters of sex-specific loci are predicted to shape the boundaries of the M/m sex-determination locus of the dengue vector mosquito *Aedes aegypti,* but the identities of these genes are not known. Identification and characterization of these loci could promote a better understanding of mosquito sex chromosome evolution and lead to the elucidation of new strategies for male mosquito sex separation, a requirement for several emerging mosquito population control strategies that are dependent on the mass rearing and release of male mosquitoes. This investigation revealed that the methylthioribulose-1-phosphate dehydratase (*MtnB*) gene, which resides adjacent to the M/m locus and encodes an evolutionarily conserved component of the methionine salvage pathway, is required for survival of female larvae.

**Results:**

Larval consumption of *Saccharomyces cerevisiae* (yeast) strains engineered to express interfering RNA corresponding to *MtnB* resulted in target gene silencing and significant female death, yet had no impact on *A. aegypti* male survival or fitness. Integration of the yeast larvicides into mass culturing protocols permitted scaled production of fit adult male mosquitoes. Moreover, silencing *MtnB* orthologs in *Aedes albopictus*, *Anopheles gambiae*, and *Culex quinquefasciatus* revealed a conserved female-specific larval requirement for *MtnB* among different species of mosquitoes.

**Conclusions:**

The results of this investigation, which may have important implications for the study of mosquito sex chromosome evolution, indicate that silencing *MtnB* can facilitate sex separation in multiple species of disease vector insects.

**Graphical Abstract:**

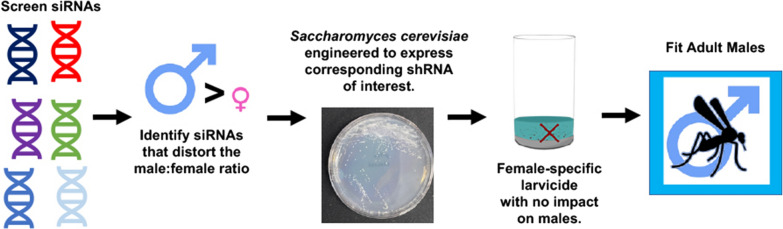

**Supplementary Information:**

The online version contains supplementary material available at 10.1186/s13071-021-04844-w.

## Background

Blood-feeding vector mosquitoes transmit disease-causing pathogens that lead to hundreds of thousands of human deaths each year. For example, Zika, yellow fever, chikungunya, and dengue result from infections with arboviruses transmitted through the bites of *Aedes* mosquitoes, including *Aedes aegypti* and *Aedes albopictus* [1]. Given poor progress in vaccine development and distribution, mosquito control is the primary mechanism for prevention of these and other mosquito-borne illnesses. Unfortunately, the emergence of insecticide resistance, concerns for the negative impacts of pesticides on the environment, and a lack of support for mosquito control programs compromise current strategies for managing mosquitoes [1].

Female mosquitoes possess sex-specific morphological, physiological, and behavioral traits, such as blood-feeding behavior, that promote the spread of disease-causing pathogens. Although genes that regulate female-specific mosquito development may represent novel targets for vector control, a majority of these genes have not yet been identified [2]. Discovery and functional characterization of female-specific genes in multiple mosquito species could reveal mechanisms that regulate female mosquito development, provide insight into the evolution of these mechanisms, and promote the development of novel mosquito control interventions. For example, gene drive strategies that involve the manipulation of sex-specific genes are being developed and characterized [3–5]. Moreover, renewed interest in the sterile insect technique (SIT), which involves the release of sterile male insects to reduce insect pest populations [6], has recently emerged in the mosquito control community [7, 8]. Broad use of SIT for mosquito control has been hampered by the need to remove female mosquitoes, which is required to reduce health and nuisance biting risks posed by the release of female mosquitoes. Removal of females is also believed to increase the efficacy of this intervention, while also reducing costs [7, 8]. Likewise, the *Wolbachia*-based incompatible insect technique (IIT), which is proving useful for mosquito population suppression [9] and can be paired with SIT [10, 11], also requires mass rearing and release of adult male mosquitoes [7, 8]. Transgenic-based population suppression approaches involving the release of insects carrying a dominant lethal (RIDL) [12, 13], as well as various emerging gene drive technologies to block pathogen transmission or to suppress mosquito populations [14], also involve the release of male mosquitoes [5]. Thus, the development and implementation of effective and affordable sex-separation technologies that can deployed worldwide are rate-limiting steps in the global introduction of numerous population-based mosquito control methods [8]. The identification of genes with sex-specific functions could permit the elucidation of novel male sex-sorting technologies that could support these interventions.

*Aedes aegypti* sex determination is regulated by a non-recombining Y-chromosome-like male-determining region, the M locus, which is present on chromosome 1 [15, 16] and contains the male-determining factor *Nix* [17]. Males, which possess one copy of the chromosome bearing the M locus and one which lacks it, have an M/m genotype, while *A. aegypti* females, which lack the male-determining locus, have an m/m genotype [18]. Clusters of loci that cause sex-specific lethal effects are believed to reside in the vicinity of the sex-determining region [19, 20]. However, the identities of these genes are not known. Characterization and sequencing of the M/m locus region had been thwarted by the repetitive nature of DNA located in this centromeric region of chromosome 1. However, recent innovations in sequencing technology generated an improved and re-annotated genome assembly that facilitated better estimation of the M/m locus, providing opportunities to study the evolution of the sex-determining chromosomes and prospects for evaluating the functional contributions of genes linked to the M/m locus [21]. In this investigation, methylthioribulose-1-phosphate dehydratase (*MtnB*), which resides adjacent to the M/m locus, was identified as a female-specific larval lethal locus.

MtnB, an evolutionarily conserved component of the methionine salvage pathway, functions to convert 5-methylthioadenosine (MTA) to methionine. This process occurs through six enzymatic reactions, the third of which, the dehydration of 5-methylthioribulose-1-phosphate (MTRu-1-P) to 2,3-diketo-5-methylthiopentyl-1-phosphate (DK-MTP-1-P), is catalyzed by MtnB [22–25]. This pathway facilitates recycling of MTA, a by-product of polyamine synthesis, back to methionine, an essential amino acid, while also serving as a useful means of recycling the sulfur present in MTA [26]. The methionine salvage pathway has also been implicated in cell death and inflammation [26–29]. In this investigation, RNAi-based silencing of *MtnB* in *A. aegypti* revealed a female-specific requirement for this gene in larvae that was exploited for the development of a scalable yeast-based oral RNAi method for production of fit adult males. Characterization of *MtnB* in several additional species of disease vector mosquitoes, including *Aedes albopictus* (vector of multiple arboviruses), *Anopheles gambiae* (malaria parasite vector), and *Culex quinquefasciatus* (lymphatic filariasis and West Nile viral vector) [1], suggested that targeting *MtnB* during larval development could facilitate male sex sorting in multiple species of disease vector mosquitoes.

## Methods

### Mosquito rearing

The *A. aegypti* LVP-IB12, *A. albopictus* Gainesville, *A. gambiae* G3, and *C. quinquefasciatus* JHB (BEI Resources, NIAID, NIH) strains were reared as described [30], except that sheep blood (purchased from HemoStat Laboratories, Dixon, CA, USA) was provided with an artificial membrane feeding system (Hemotek Limited, Blackburn, UK).

### Larval soaking

Custom small interfering RNAs (siRNAs) corresponding to mosquito *MtnB* genes (Table 1) were selected using the Integrated DNA Technologies (IDT) Custom Dicer-Substrate siRNA (DsiRNA) tool [31]. Custom siRNAs (see Table 1 for further information), as well as a control siRNA lacking a known target in mosquitoes [32], were purchased from IDT (Coralville, IA, USA) for use in larval soaking screening experiments that were performed in duplicate using the method of Singh et al. [33]. For each soaking experiment, 20 first instar larvae (L1) were soaked in 0.5 µg/µl control or experimental siRNA (Table 1) for 4 h and then reared as described [34]. Soaking data were statistically evaluated using the chi-squared test to identify statistically significant differences between the observed and expected survival of female and male mosquitoes, assuming expected 1 male:1 female ratios on the basis of laboratory observations of the mosquitostrains when reared in the laboratory insectary.

**Table 1:** siRNA target sequences, corresponding genes, and resulting male:female sex ratios following treatments

siRNA	Target sequence	Corresponding genes	Species	siRNA soaking treatment Males: Females (*n*)	Male mortality *P* value	Female mortality *P* value
496	GAACAUGCUAUGAAAGAAUAUCCUG	*AAEL011830*	*A. aegypti*	23:12 (40)	1.0000	0.0033
529	GCAUCAAGCUUGAUGAUGAAAUUUA	GAPW01003631.1, Aa-53178 mRNA	*A. albopictus*	10:4 (20)	1.0000	0.0108
523	CGUGGAUGCAUGAUAAUCGAAUAGU	*AGAP000470*	*A. gambiae*	28:12 (40)	1.0000	0.0033
534	AGAAUAUCGAUGGAGAUGAUCUGCA	*CPIJ011357*	*C. quinquefasciatus*	25:7 (40)	1.0000	0.0001

siRNAs and corresponding target sequences/genes in the indicated species, and the altered male:female ratios resulting from siRNA soaking treatments are indicated (*P* values correspond to Fisher’s exact tests)

### Production of yeast interfering RNA larvicides

Yeast strains were prepared as described [35] through cloning of custom shRNA expression cassettes corresponding to target sequences of interest (Table 1; synthesized by Invitrogen, Carlsbad, CA, USA) into the *pRS426 GPD* shuttle vector [36]. After confirming the clones through restriction digestion and sequencing, the resulting plasmids were used to transform *S. cerevisiae CEN.PK* yeast (genotype *MATa/α ura3-52/ura3-52 trp1-289/trp1-289 leu2-3_112/leu2-3_112 his3 Δ1/his3 Δ1 MAL2-8C/MAL2-8C SUC2/SUC2* [37]. Transformants were selected based on growth on minimal media lacking uracil. shRNA expression was confirmed through PCR conducted as described [38] using the following primers: forward 5′-CAGGATATTCTTTCATAGCATGTTC-3′ (specific to the 3′ end of the *MtnB* hairpin) and reverse primer 5′-TCCTTCCTTTTCGGTTAGAGC-3′ (which corresponds to the terminator sequence). An image of the PCR products visualized with ethidium bromide staining (Additional file 1: Fig. S1) was edited using Adobe Photoshop 2021 software (to remove non-relevant lanes and to label the figure). Following strain confirmation, dried inactivated yeast interfering RNA was prepared for use in larvicide assays as described [35].

### Yeast interfering RNA larvicide screening

Yeast interfering RNA larvicides were evaluated through larvicide assays that conformed to the World Health Organization (WHO) [39] guidelines, which were performed as previously described [35]. In summary, 20 L1 larvae were placed in 500 ml plastic cups containing 50 ml of distilled water and 50 mg of dried inactivated yeast (either experimental larvicidal yeast or yeast prepared from a control interfering RNA strain [34]). The emergence rates and sexes of the resulting adults were recorded in each of nine replicate trials that were performed for each control and larvicidal yeast treatment. Data were evaluated using the chi-squared test to identify statistically significant differences between the observed and expected 1 male:1 female ratios, female survival, and male survival for each treatment.

### qRT-PCR assays

*MtnB* silencing was confirmed through qRT-PCR assays that were performed as described [40]. In summary, TRIzol Reagent (Invitrogen, Carlsbad, CA, USA) was used according to the manufacturer’s instructions to prepare total RNA from 20 pooled third instar larvae that had fed on either control or MtnB.496 yeast throughout larval development. cDNA was prepared from total RNA extracts using the High Capacity RNA to cDNA Kit (Applied Biosystems, Foster City, CA, USA) according to the manufacturer’s instructions. Real-time PCR quantification assays were performed using an Applied Biosystems Step One Plus Real-Time PCR System in conjunction with the Power SYBR Green PCR Master Mix as described by the manufacturer (Applied Biosystems, Foster City, CA, USA). The following primer sets were used in these reactions: forward 5′-GGC AAT GGC TGA ATG TTA CG-3′ and reverse: 5′-GTG ATT GGA ATC AGA ATT GAC TTA-3′. Amplification of *rpS17*, an internal standard for data normalization, was performed as described [40] with the following primers: forward 5′ AGA CAA CTA CGT GCC GGA AG 3′ and reverse 5′ TTG GTG ACC TGG ACA ACG ATG 3′. PCR amplifications were performed in six replicate wells in each of two separate biological replicate trials, and results were quantified by standardizing reactions to *rpS17* transcript levels using the ΔΔCt method as described [40]. Data were statistically evaluated with Student’s *t*-test.

*MtnB* developmental transcript expression was quantified in two separate pools of ten untreated individuals for L1 larvae, L2 larvae, L3 larvae, L4 larvae, male pupae, and female pupae (note that the pupal time point is the first stage in which *A. aegypti* sexes are visibly discernable). Total RNA from the separate pools was prepared and analyzed as described above. Results were quantified by standardizing reactions to *rpS17* transcript levels using the ΔΔCt method as described [40]. Setting the expression level of L4 larvae to one permitted standardization and comparison of qRT-PCR results across the different stages. Transcript expression data were analyzed using analysis of variance (ANOVA).

### Male life history trait assessments

Life history traits were evaluated as described by Hill et al. [41]. In these assays, males that had been reared on control interfering RNA or MtnB.496 yeast (*n* = 52 combined from two separate biological replicate trials) were mated as individuals to wild-type females, which had been reared as described [30]. Mating success was measured by confirming that females had laid eggs; if females failed to lay eggs, mating success/failure was further assessed through dissection of the spermatheca to confirm the presence or absence of sperm. Mating data were analyzed with the chi-squared test. For successful matings (*n* = 51 females mated to control males and *n* = 49 females mated to Mtn.496-treated males), the number of eggs laid per individual female (fecundity) and number of larvae that hatched (fertility) were recorded. Fertility and fecundity data were analyzed with Student’s *t*-test.

### Mass-rearing trials

Two hundred L1 LVP-IB12 larvae were placed in 34 × 25 × 4 cm trays (1426B, Bioquip, Rancho Dominguez, CA, USA) bearing 1 L of distilled water. Per Zhang et al*.* [42], larvae were cultured using a mass-rearing diet that consisted of a slurry of 250 mg bovine liver powder (MP Biomedicals, Santa Ana, CA, USA) and 150 mg of ground shrimp (Tetra GMBH, Melle, Germany) mixed with 10 ml water, but with the brewer’s yeast component of the diet [42] replaced with yeast interfering RNA. The yeast interfering RNA component (control or Mtn.496) was combined with the liver powder and shrimp slurry and fed to larvae as follows: 100 mg at L1, 100 mg at L2, 100 mg at L3, and 200 mg at L4. Each day, larval trays were examined for pupae, which were removed, sorted by sex, and counted. Mortality and sex ratio distortions were evaluated in three biological replicate trials, each with two replicate trays of larvae fed using diets prepared with control interfering RNA or MtnB.496 yeast. Statistically significant differences between the observed and 1:1 expected survival levels for males and females were evaluated using the chi-squared test. The fitness of surviving males was ascertained through comparisons of wing lengths, which were measured as described [43]. Wing length data were evaluated with Student’s *t*-test.

## Results

### Silencing MtnB during *A. aegypti* larval development selectively kills female mosquitoes

**Fig. 1:**
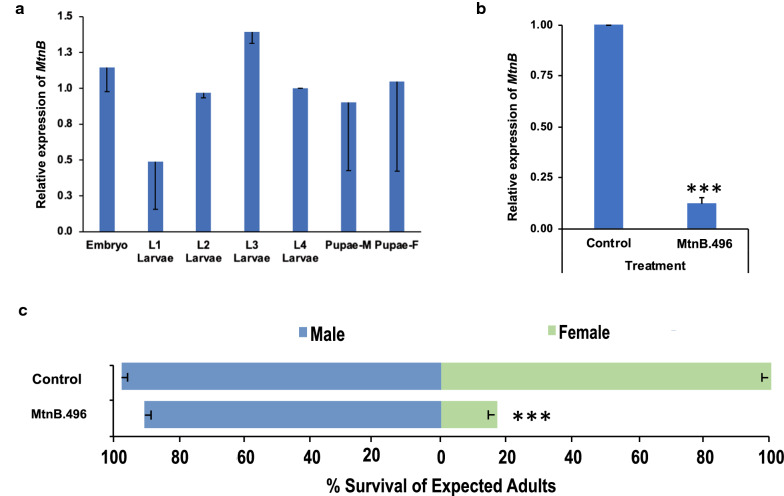
Yeast larvicide MtnB.496 induces female-specific mortality. **a** *MtnB* transcript is expressed throughout larval development (L1 = first instar, L2 = second instar, L3 = third instar, L4 = fourth instar, Pupae-M = male pupae, Pupae-F = female pupae; error bars denote standard deviations; transcript levels relative to those of L4 larvae are displayed). **b** Silencing of *MtnB* following larval consumption of MtnB.496 yeast was confirmed through qRT-PCR (***P < 0.001, Student’s *t*-test; error bars represent standard deviation). **c** Oral consumption of MtnB.496 yeast during larval development resulted in significant female larval mortality (***P < 0.001, chi-squared test), but did not impact male survival (*P* > 0.05, chi-squared test); larval consumption of control interfering RNA strain yeast did not significantly impact survival of female (*P* > 0.05, chi-squared test) or male (*P* > 0.05, chi-squared test) larvae. Data, which were compiled from nine replicate container trials per condition (with each container bearing 20 larvae), are represented as mean survival through adulthood, and error bars represent standard errors of the mean

Given that the M/m locus is believed to be tightly linked to developmental genes that confer sex-specific effects in *A. aegypti*, *MtnB,* which is located on chromosome 1 and flanks the M/m locus [21, 44], was hypothesized to function as a potential sex-specific lethal gene. *MtnB* was silenced through larval soaking with siRNA #496, which corresponds to the *MtnB* transcript. Treatment with siRNA #496 resulted in significant female mortality yet had no significant impact on male survival (further details, including *P* values, are provided in Table 1). Given the results of this brief siRNA soaking-mediated transient gene silencing assay, *S. cerevisiae,* which was recently developed as a system for expression and sustained delivery of interfering RNA to mosquito larvae, was used to express a short hairpin RNA (shRNA) corresponding to siRNA #496. shRNA expression in this yeast strain, hereafter referred to as MtnB.496, was confirmed through PCR amplification of cDNA corresponding to the shRNA transcript (Additional file 1: Fig. S1). Dried inactivated MtnB.496 yeast was fed to larvae throughout larval development. Larvae were also reared on yeast that expresses an shRNA with no known target in mosquitoes [34] for comparison. *MtnB* transcripts, which are detected during larval and pupal development (Fig. 1a), are silenced in larvae that consume MtnB.496 yeast (Fig. 1b; 88 ± 4% reduction in transcript levels in comparison to larvae reared on control interfering RNA yeast; *t*-test, *P* < 0.001). Larval consumption of MtnB.496 yeast, but not control interfering RNA yeast, resulted in significantly higher male:female ratios among the surviving mosquitoes (Fig. 1c; *χ*^2^ = 31.387, *P* < 0.0001). Although no significant impact on male survival was observed following consumption of MtnB.496 yeast (*χ*^2^ = 3.214, *P* > 0.05), only 13 ± 2% of expected adult female mosquitoes survived, with a majority of animals dying as late larvae or early pupae (*χ*^2^ = 128.571, *P* < 0.0001).

### Scaled production of fit adult *A. aegypti* male mosquitoes

**Fig. 2:**
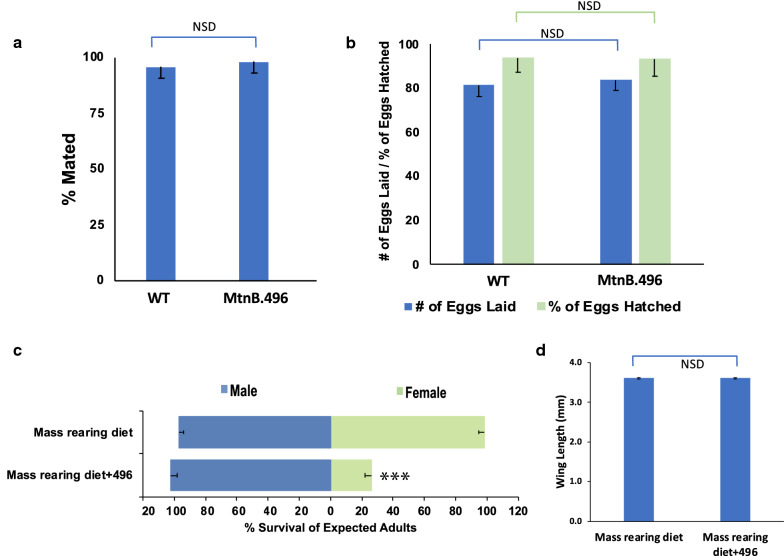
MtnB.496 yeast facilitates scaled production of fit *A. aegypti* males. Larval consumption of MtnB.496 yeast does not significantly impact adult male mating capacity (**a**, *P *> 0.05, chi-squared test, *n* = 49), the number of eggs laid by females that mate with these males (**b**, *P* > 0.05, Student’s *t*-test), or the percentage of larvae hatching from these eggs (**b**, *P* > 0.05, Student’s *t*-test). Incorporation of MtnB.496 yeast into the diet used for mosquito mass rearing induced significant female mortality (**c**, ***P < 0.001 vs. mass-rearing diet, chi-squared test) yet did not significantly impact male survival (**c**, *P* > 0.05 vs. mass-rearing diet, chi-squared test; data were compiled from four replicate containers per condition, each bearing 500 larvae) or wing size (**d**, *P* > 0.05 vs. mass-rearing diet, *t*-test; *n* = 50 wings/treatment), which correlates with fitness. Error bars represent SEM in all panels. NSD: Not significantly different

To confirm that the impacts of MtnB.496 larvicide are specific to female larvae, life history traits were assessed in adult male mosquitoes that had consumed the yeast throughout larval development. The percentage of treated males that mated with wild-type females was not significantly different than adult males that were reared on control yeast interfering RNA larvicides (Fig. 2a, *χ*^2^ = 0.230, *P* > 0.05). When the MtnB.496-treated males mated with wild-type female mosquitoes, no significant differences in female fertility (Fig. 2b, *t* = −1.925, *P* > 0.05) or fecundity were observed (Fig. 2b, *t* = 0.698, *P* > 0.05). These findings indicated that MtnB.496 yeast larvicide treatment did not produce any detectable effects on male fitness in the assays conducted.

To assess whether the RNAi-based yeast larvicides could also facilitate scaled production of males, a larval diet and feeding regimen employed at *Aedes* mass-rearing facilities [42] was modified through replacement of the nutritional yeast component of the diet with dried inactivated yeast larvicide MtnB.496. The modified diet was tested on *A. aegypti* larvae grown in mass-rearing trays. Under these conditions, the larvicides continued to induce significant *A. aegypti* female mortality (Fig. 2c, *χ*^2^ = 1275.764, *P* < 0.0001) with no impacts on male survival (Fig. 2c, *χ*^2^ = 0.5777, *P* > 0.05). The fitness of male survivors, which was estimated through measurement of wing lengths, a correlate of body size and fitness, was not significantly different than that of males reared on the standard mass-rearing diet (Fig. 2d, *t* = 1.031, *P* > 0.05).

### MtnB orthologs are female-specific lethal genes in multiple species of disease vector mosquitoes

*MtnB* orthologs have been identified in *A. gambiae* and *C. quinquefasciatus,* and BLAST searches identified an ortholog in *A. albopictus* (Table 1). RNAi-mediated targeting of these genes through brief soaking treatments in siRNAs #529 (corresponds to *A. albopictus MtnB*), #523 (corresponds to *A. gambiae MtnB*), and #534 (corresponds to *C. quinquefasciatus MtnB)* resulted in significant female-specific larval death, yet did not impact male survival through adulthood (Table 1, statistics provided in table). Yeast interfering RNA larvicide strains that express shRNAs corresponding to siRNAs #529, #523, and #534 were therefore constructed and are hereafter referred to as strains MtnB.529 (targets *A. albopictus),* MtnB.523 (targets *A. gambiae),* and MtnB.534 (targets *C. quinquefasciatus*).

**Fig. 3:**
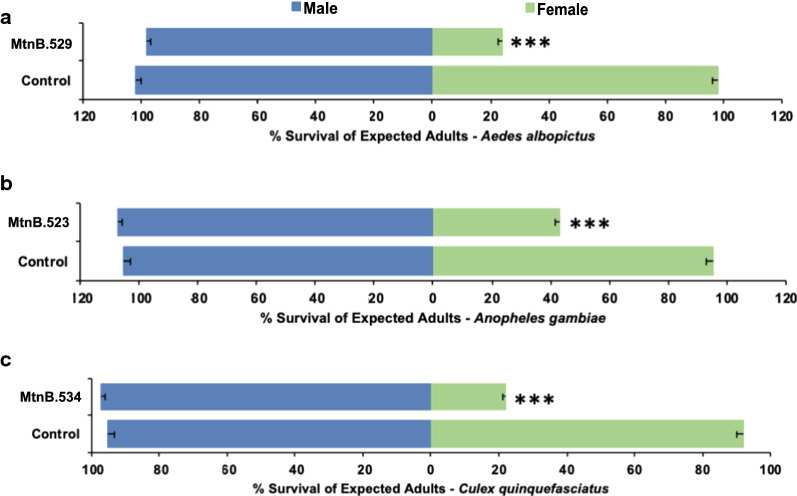
MtnB is required for female larval survival in multiple species of mosquitoes. Larval consumption of yeast interfering larvicides corresponding to the *A. albopictus* (**a**), *A. gambiae* (**b**), and *C. quinquefasciatus* (**c**) *MtnB* genes induce significant female-specific mortality in each species (***P < 0.001, chi-squared test), but have no impact on male survival (**a–c**, *P* > 0.05, chi-squared test). Controls in **a–c** were larvae fed with yeast expressing an interfering RNA with no known target in mosquitoes, which had no significant impact on female or male survival in any of the three mosquitoes species (**a**–**c**, *P* > 0.05, chi-squared test). Data, which were compiled from nine (**a** and **c**) or six (**b**) replicate container trials per treatment (with each container bearing 20 larvae), represent mean survival through adulthood, with error bars corresponding to SEM

Larval consumption of MtnB.529 by *A. albopictus* larvae resulted in significantly higher than expected male:female ratios (Fig. 3a; *χ*^2^ = 24.202, *P* < 0.0001). Only 24 ± 1% of expected *A. albopictus* female mosquitoes survived (Fig. 3a, *χ*^2^ = 101.829, *P* < 0.00001), but no significant impact on male survival was observed (Fig. 3a, *χ*^2^ = 2.067, *P* > 0.05). Likewise, MtnB.523 resulted in significantly higher male:female ratios among surviving *A. gambiae* adults (Fig. 3b; *χ*^2^ = 7.453; *P* < 0.01). Although MtnB.523 had no significant impact on the survival of males through adulthood (*χ*^2^ = 0.004; *P* > 0.05), only 43 ± 2% of expected *A. gambiae* female mosquitoes survived (Fig. 3b, *χ*^2^ = 37.551, *P* < 0.0001). Finally, significantly higher than expected male:female ratios were also observed among *C. quinquefasciatus* adult survivors following larval consumption of MtnB.534 (Fig. 3c; *χ*^2^ = 28.751, *P* < 0.0001), a difference that coincided with only 21 ± 1% of expected *C. quinquefasciatus* adult female emergence (Fig. 3c; *χ*^2^ = 95.890; *P* < 0.00001), with no significant corresponding changes in male survival (Fig. 3c; *χ*^2^ = 3.051, *P* > 0.05).

## Discussion

### Implications for understanding the evolution of sex chromosomes in mosquitoes

Sex chromosome evolution is believed to occur in several stages [45–47], starting with a homologous pair of autosomes that acquire sex-determining loci. A proto-Y chromosome, which has a male fertility locus (M) and a dominant female suppressor locus (Su^F^), as well as a proto-X chromosome carrying a female fertility locus (F) and a male sterility locus (m) form. Suppressed recombination in the sex-determining region evolves, eventually spreading along the proto-sex chromosomes, which accumulate transposable elements and non-coding sequences, undergo chromosomal rearrangements, and accumulate sexually antagonistic genes with different alleles that differentially benefit either males or females. The proto-sex chromosomes eventually evolve into heteromorphic X and Y chromosomes, with the Y chromosome eventually degenerating and reducing in size [45–47].

Sex chromosomes in different mosquito species have progressed to varying stages of this evolution. Culicines, including *Aedes* and *Culex* mosquitoes, possess homomorphic sex chromosomes in which the sex-determination alleles are linked to the smallest homomorphic first chromosome [48]. In *A. aegypti,* the homomorphic sex chromosomes have evolved into proto-sex chromosomes bearing a sex determining M/m locus [15, 16]. The M locus at the proto-Y chromosome contains the male-determining factor, *Nix* [17], which directs male-specific splicing of the sex chromosome gene *doublesex (dsx)*. In females, which have two copies of the proto-X (m) chromosome, the female-specific Dsx^F^ form regulates ovary development and fertility [43]. Although a sex-differentiated region of suppressed recombination extends ~ 100 Mb beyond the M/m locus [21, 49, 50], heteromorphic X and Y chromosomes have not yet evolved in *A. aegypti.* Likewise, *A. albopictus* also possesses homomorphic sex chromosomes, and the *A. albopictus Nix* gene was identified as a strong candidate for a male-determining factor in this species [17, 51, 52]. In contrast, *A. gambiae* has already evolved separate X and Y chromosomes, with the *Yob* gene functioning as a male-determining factor on the Y chromosome [53]. Despite different mosquito species having advanced to various stages of sex chromosome evolution, this investigation functionally verified that *MtnB* is required for survival of female larvae in *A. aegypti* (Figs. 1c, 2c), *A. albopictus* (Fig. 3a), *A. gambiae* (Fig. 3b), and *C. quinquefasciatus* (Fig. 3c) mosquitoes. These findings suggest that loci which cause sex-specific lethal effects and shape the boundaries of the sex-determination locus were accumulating on the sex chromosomes prior to separation of the culicine and anopheline mosquitoes. Future studies will be directed toward understanding the nature of this female-specific requirement during larval development.

Krzywinska et al. [20] discuss possible mechanisms underlying the sex-specific lethality they observed in *A. aegypti* that had inherited sex chromosomes produced through male meiotic recombination events that occurred between the sex-determination locus and a linked enhanced green fluorescent protein (EGFP)-marked transgene. An explanation which is consistent with the *MtnB* silencing results presented here proposed that such recombination events could lead to the loss of a haploinsufficient sex chromosome gene for which two copies are required in m/m females. If *MtnB* is haploinsufficient in females, any female that inherits a recombinant m chromosome lacking a copy of the *MtnB* gene will die, an event that is sometimes referred to as “suppression of recombination” because resulting female offspring are not observed in adult populations. Likewise, as observed in this investigation, silencing *MtnB* using RNAi-based yeast larvicides can also result in female death. These silencing results suggest that the presence of haploinsufficient genes adjacent to the sex-determination locus may have helped to shape the boundaries of the sex-determination region during mosquito sex chromosome evolution, as previously suggested [20].

### Sex-separation applications

Papathanos et al. [54] recommend that sex-sorting methodology should be simple, stable, and produce competitive males, ideally through female elimination during development. The yeast female-specific larvicide system described herein meets these criteria (Fig. 2). It is also recommended that the system be cost-effective, scalable, and suitable for implementation in remote or resource-limited regions of the world [54], criteria that would be met through the use of yeast, which is routinely produced at commercial scale by the food industry for worldwide distribution. Moreover, the demonstrated ability to seamlessly incorporate yeast into a standard mass-rearing diet (Fig. 2c) makes this system simple to implement. Sex-sorting technology should meet regulatory standards, ideally requiring no modification of existing regulatory protocols that may have already been acquired for the mosquito technology under development. It is also important that the technology for elimination of females be conditional, so as not to interfere with strain maintenance [54]. The yeast sex-sorting system meets these criteria, as it induces no permanent changes to the mosquito genome, but acts through conditional gene silencing during female development. The use of yeast RNAi-based larvicides would therefore circumvent the need to further genetically manipulate existing mosquito strains developed for population control strategies. Furthermore, the yeast would be used only during the larval stages and would be heat-killed prior to use indoors at mass-rearing facilities. This may mitigate a need for modification of existing regulatory permits previously obtained for male mosquito releases, a prospect that can be further discussed with the appropriate regulatory bodies. Thus, sex-specific yeast interfering RNA technology meets many of the criteria for a useful sex-separation system [54]. Future studies will be directed toward scaled industrial-sized production of yeast interfering RNA larvicides for distribution of yeast to mass-rearing mosquito facilities worldwide.

In this investigation, replacement of the nutritional yeast component of a larval mass-rearing diet [42] resulted in female-specific larval killing, facilitating production of fit male mosquitoes at a ratio of 5 male:1 female mosquitoes (Fig. 2d). Silencing *MtnB* did not eliminate all females (Fig. 2b, c) and this yeast strain could not likely be used as a stand-alone strategy for male mosquito production. It would be interesting to explore methods for further improving the female-killing capacities of the yeast strains (i.e. increased shRNA expression levels, stability, or uptake). We hope to explore this in future studies, as a stand-alone strategy that generates > 99% male separation, as observed through use of a sophisticated camera system recently developed for *A. aegypti* [9], would be preferable. Although the automated camera system is highly effective, it is not yet clear if such technology can be deployed worldwide or adapted for use is all mosquito species. In such instances, and particularly when resources are limited, replacing nutritional yeast with larvicidal yeast during the mass-rearing process could be used in combination with existing sex-separation technologies, particularly manual separation strategies, which are highly labor-intensive [8].

Female-specific yeast larvicide technology could particularly benefit the development and implementation of population-based control strategies for *C. quinquefasciatus* (Fig. 3c)*,* in which sex-sorting methods other than physical separation have yet to be established [7, 8]. The female-specific mortality levels observed in the current investigation were relatively lower in *A. gambiae* with respect to the other mosquitoes evaluated (Fig. 3). However, given the conservation of *MtnB* in many species of *Anopheles* mosquitoes [44], it may nevertheless still be worth exploring the potential for using *MtnB* silencing to sex other *Anopheles* species. For example, the importance of developing sex-separation strategies that could be implemented in malaria vectors such as *Anopheles arabiensis* [55] and *Anopheles stephensi* [56] has been discussed [44], and *MtnB* is conserved in both species, as well as several other anophelines. It may therefore be helpful to pair a yeast larvicide diet with the manual or visual sorting strategies currently employed in these *Anopheles* mosquitoes [7]. One could also envision pairing the RNAi yeast diet sex-separation approach with various transgenic sex-sorting strains that have been generated for several species, including female lethal strains or transgenic fluorescent marker strains [57, 58] that facilitate automated sorting. Likewise, yeast feeding could be combined with imaging-based sorting strategies [9] and automated pupal size estimators [59], or paired with feeding of toxicant-infused blood meals to females [60]. Such combined approaches may better permit the > 99% removal of females that is generally accepted as a minimal requirement for male-only release control strategies [7].

## Conclusions

These studies have uncovered a conserved female-specific larval requirement for *MtnB,* which encodes a component of the methionine salvage pathway, in distantly related mosquitoes. The results of this investigation may have important implications for the study of mosquito sex chromosome evolution, and it will be interesting to further investigate this topic in future studies. It is anticipated that female-specific yeast interfering RNA technologies that capitalize on the conserved female-specific requirement for *MtnB* function in mosquito larvae could permit mass production of fit males, thereby facilitating emerging population-based strategies for control of multiple disease vector mosquito species.

## Supplementary Information

**Additional file 1: Fig. S1.** Confirmation of shRNA expression in recombinant yeast strain MtnB.496. PCR reactions performed with primers corresponding to the MtnB.496 shRNA transcript generated a ~ 100 bp amplicon (see DNA standard at far left; cDNA template was prepared from MtnB.496 yeast total RNA). Negative control PCR reactions included an amplification with cDNA prepared from non-transformed yeast (marked by – sign) and a reaction with no cDNA added (marked by knot symbol). A representative ethidium bromide-stained agarose gel from one of two comparable biological replicate experiments is shown.

## Data Availability

All data generated or analyzed during this investigation are included in this manuscript. Yeast strains and corresponding plasmids generated during the course of this study will be made available upon satisfactory completion of a material transfer agreement with Indiana University and following procurement of required import permits by the requesting party.

## Abbreviations

DK-MTP-1-P: 2,3-Diketo-5-methylthiopentyl-1-phosphate
MTA: 5-Methylthioadenosine
MTRu-1-P: 5-Methylthioribulose-1-phosphate
*Dsx*: *doublesex*
L1: First instar larvae
IDT: Integrated DNA Technologies
LVP-IB12: Liverpool-IB12
MtnB: Methylthioribulose-1-phosphate dehydratase
shRNA: Short hairpin RNA
siRNA: Small interfering RNA
