# Supplementary figures and images for: A conserved female-specific larval requirement for MtnB function facilitates sex separation in multiple species of disease vector mosquitoes
Source: Parasit Vectors. 2021 Jun 26;14:338. doi: 10.1186/s13071-021-04844-w (PMC8234664; doi:10.1186/s13071-021-04844-w)

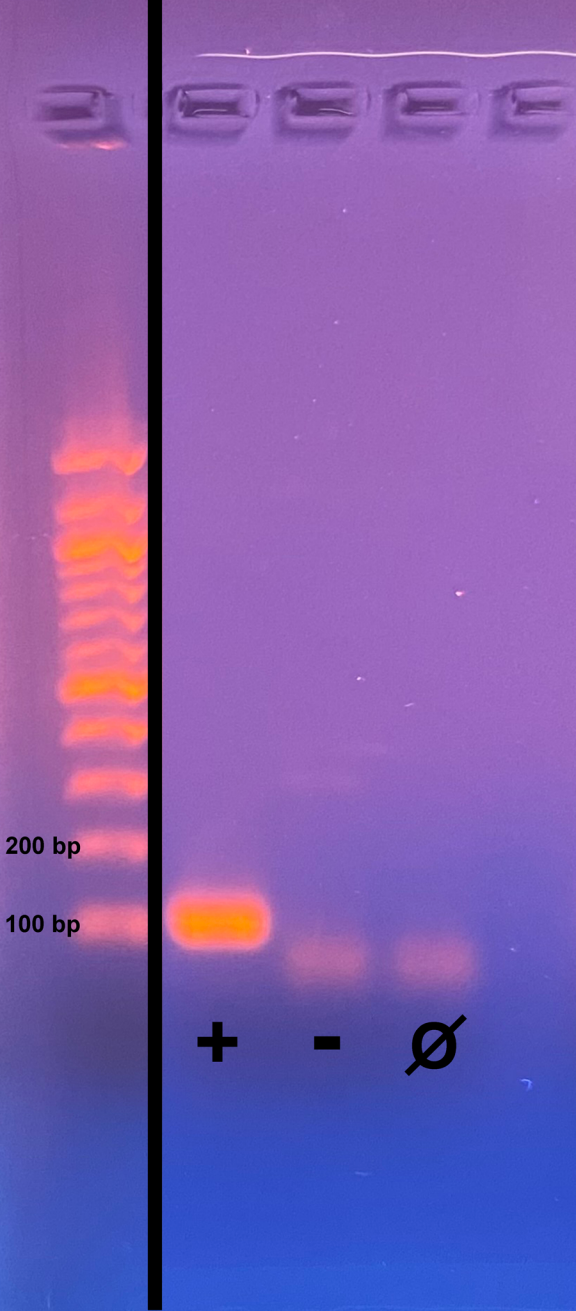

Supplement: Supplementary file 1 — Additional file 1: Fig. S1. Confirmation of shRNA expression in recombinant yeast strain MtnB.496. PCR reactions performed with primers corresponding to the MtnB.496 shRNA transcript generated a ~ 100 bp amplicon (see DNA standard at far left; cDNA template was prepared from MtnB.496 yeast total RNA). Negative control PCR reactions included an amplification with cDNA prepared from non-transformed yeast (marked by – sign) and a reaction with no cDNA added (marked by knot symbol). A representative ethidium bromide-stained agarose gel from one of two comparable biological replicate experiments is shown. [file 13071_2021_4844_MOESM1_ESM.pdf]
